# Supplementary material for: Are carbon emissions trading and green financial instruments synergistic? -Comprehensive quantitative research based on content analysis
Source: PLoS One. 2024 Mar 7;19(3):e0298601. doi: 10.1371/journal.pone.0298601 (PMC10919601; doi:10.1371/journal.pone.0298601)
Supplement: S1 Data — (ZIP) [file pone.0298601.s001.zip › Data/地级市Z_F工作报告.zip!/╜¡╬≈/╔╧╚─╩╨/└┤╫╘╣1⁄2╓┌║┼í╛┬φ┐╦╩2╛▌═°í┐_2014.docx]

各位代表：

现在，我代表市人民政府，向大会报告政府工作，请予审议，并请市政协委员和列席会议的同志提出意见。

一、2013年工作回顾

2013年，是极不平凡的一年。面对极为错综复杂的国内外形势，在省委、省政府和市委的坚强领导下，全市上下深入贯彻落实党的十八大、十八届二中、三中全会、习近平总书记系列讲话和省委十三届七次全会精神，紧紧围绕市三届人大三次会议确定的目标任务，以提高经济增长质量和效益为中心，深入实施“八大战略”，强力推进“八个支持”，扎实做好稳增长、调结构、促改革、打基础、优生态、惠民生等各项工作，努力克服前进中的困难和挑战，取得了农业生产“十连丰”、农民收入“十连增”、县县财政收入过十亿元、主要经济指标全部实现两位数以上增长的成绩，保持了经济社会稳中有进、稳中向好的发展态势。

一年来，主要做了以下工作：

(一)全力化解国际国内贸易风险，避免了经济大起大落

政府采取财政政策引导、发行企业债券担保等果断调控措施，在全国率先顶住了欧美对中国光伏产业最严格的“双反”制裁，不仅保住了上饶全国光伏产业基地的位子，而且使晶科能源在全国光伏产业中率先走出困境，实现盈利。针对多数企业产品价格下跌尤其是铜产品价格大幅下跌、大部分中小企业经营困难的情况，政府强化了对经济运行的调度，采取降低生产成本、多渠道融资等多种有效政策调控措施，切实帮助企业渡过难关，有力地支撑了全市经济持续健康发展。全市实现生产总值1401亿元，增长10.0%。完成财政总收入232亿元，增长15.8%，其中公共财政预算收入164.4亿元，增长22.6%。规模以上工业增加值507亿元，增长12.9%。社会消费品零售总额485亿元，增长13.6%。

(二)强力推进新型工业化，做大做强经开区迈开新步伐

制订了新三年加速推进工业化的实施意见，立足工业园区，抓项目、增投入、扩总量、提效益，重点推进战略性新兴产业发展和传统产业改造升级。开工、在建投资亿元以上工业项目124个，其中列入省重点调度项目21个。完成工业投资680.9亿元，较上年增加97.9亿元。新增规模以上工业企业158户。新增主营业务收入过200亿元园区1个、过100亿元园区1个。工业园区实现税收123.6亿元，增长58.9%，占全市财政总收入的比重达到53.3%。战略性新兴产业实现增加值237.5亿元，增长13%。

在全力支持经开区做大做强政策推动下，经开区快速发展。编制完成了100平方公里总体规划。沪昆高速经开区互通、两条新辟园区主干道、污水处理厂等一批重大基础设施基本建成。新引进亿元以上工业项目20个，其中投资过30亿元项目3个。饶商回归产业园吸引饶商投资26亿元。经开区实现工业增加值119.4亿元，增长27.4%;完成工业主营业务收入576.3亿元，在全省排名前移一位。

(三)大力推进农业产业化，建设全国优质农产品种养加工基地呈现新亮点

出台了加快农业产业化发展的实施意见，以示范区和龙头企业为抓手，大力推进农业产业化。上饶现代农业科技园建成全省第四个国家级农业科技园。全市新增规模以上农业企业87家、省级农业龙头企业14家、农民专业合作社1511家，新发展家庭农场68家，创建省级现代农业示范区6个。市级以上农业龙头企业实现销售收入270亿元，带动农户户均增收2142元。改造中低产田38.5万亩，新增、改善灌溉面积45万亩，建设高标准农田22.4万亩。伦潭水利枢纽主体工程基本完工。

在全力支持鄱余万建设全国优质农产品种养加工基地政策推动下，大规模的基地建设顺利推进。鄱阳县推进了高产油茶、万亩湿地松速丰林、花卉苗木等一批优质基地建设，连续十年获得“全国粮食生产先进县”，被列为全国粮食增产模式攻关县。余干县建成了10万亩优质高产水稻、3000亩枫树辣椒、全国最大的万亩连片芡实和6000亩连片生态泥鳅等种养基地。万年县成功创建国家农业产业化示范基地，建立花卉苗木基地3万亩，新增贡米基地5万亩。

(四)加速推进旅游、商贸、物流、金融业发展，婺源、三清山创建全国一流景区掀起新高潮

大力促进旅游业发展升级，新增国家4A级景区3个，推进了龟峰、神农源、灵山、大茅山、铜钹山等景区的开发，全年旅游接待人数5400万人次，旅游综合收入433.5亿元，继续保持全省第一。加快建设现代商贸物流体系，成功争取到国家中小商贸流通企业服务体系建设试点城市;引进了海西(上饶)胜记国际物流城、万达广场、万年青城等特大型物流和特大型商业综合体项目;坑口铁路物流专用线(上饶西货站)已与南昌铁路局签约;全市建成大型商业综合体12个，交易额超亿元的市场达42个;上饶电子商务产业园被评为全省电子商务示范基地。加快推进金融保险业发展，新增设银行业金融机构市级分行1家，一级支行6家;全市金融机构人民币存款余额1665.2亿元，社会融资余额1558.7亿元，存款、融资均居全省前列;保险业实现保费收入27亿元。

在全力支持婺源、三清山建设全国一流景区政策推动下，两地创建工作亮点纷呈。婺源县新增国家4A级景区和4A级乡村旅游点各1个，并成功争取国家生态旅游示范区、国家智慧城市试点县、美丽乡村建设试点县和“中国最美的六大乡村古镇”等品牌，全年接待游客突破1000万人次，旅游综合收入达51.8亿元。三清山被列为全省首个旅游发展示范景区，被评为“2013年大中华区最美自然生态旅游景区”和“中国最美的五大峰林”，全年接待游客突破800万人次，旅游综合收入达58.7亿元。

(五)全力推进重大基础设施建设，扩大改革开放合作平台建设有了新支撑

全市完成500万元以上固定资产投资1165亿元，增长17.9%。三清山机场主体工程全面开工。杭南长、合福两条高铁进入铺轨阶段。九景衢铁路启动征迁工作。上万高速公路项目工程可行性研究报告编制完成。德上高速三清山连接线基本建成，德上高速怀玉山连接线开工建设。福州港上饶码头加快建设，万年港综合码头基本建成。“西气东输”二线和中石化江西成品油管道二期工程进展顺利。完成各类农村公路1600公里、桥梁28座，建设农村客运中心5个。新改建220千伏变电站5个、110千伏变电站8个。4G网络覆盖到县城。

在省委、省政府的高度重视和大力支持下，我市扩大开放合作“发挥向东桥头堡作用，加快建设光伏、光学、先进装备制造产业基地和全国旅游强市，打造我省东部的重要增长极”上升为省级区域发展战略。改革创新力度进一步加大，完成了中心城市污水处理厂国有化改革，推动了基层农信社改制组建农商行工作，启动了市城投公司、市国资公司政府融资平台改革，平稳推进了“营改增”试点改革、事业单位分类改革、公立医院改革、基层农技推广体系改革、中小学教师职称制度改革等工作。对外开放取得新成果，全年实际利用外资7.6亿美元，增长12.2%;利用省外5000万元以上项目实际进资370.3亿元，增长21.1%;出口总值30.5亿美元，增长16.1%;上饶至宁波“五定班列”发运集装箱2.7万标箱，增长45.9%。非公经济快速发展，新增私营企业5414户、个体工商户2.5万户，总数分别达到2.8万户、17.7万户;非公经济完成增加值933亿元，占生产总值的比重达66.6%。

(六)加快推进新型城镇化，打造“1+5”信江河谷城镇群展开新篇章

确立了以中心城市建设为引领、统筹城乡发展的新路子。全市开工城建项目722个，完成投资366亿元，其中中心城市开工重点城建项目21个，完成投资48.7亿元。全市建成区面积294.1平方公里，比上年增加31平方公里，其中中心城市建成区面积78.9平方公里，比上年增加8平方公里。重点推进的中心城市“十大工程”进展顺利，上饶南大道、凤凰大道西延伸段、广信大厦、三江水系改造和云碧峰入口景观工程等项目基本建成，上饶北大道、凤凰大道东延伸段、老火车站片区综合改造、保障性安居工程、城市供水水源引水工程、上饶师范迁建、三甲医院及康复中心、天佑大道、稼轩大道、市民创业文化公园、陆羽公园、万力时代广场等项目推进加快，天然气管道已与省对接通气，新火车站综合交通枢纽、污水处理厂改扩建西迁等项目陆续开工。城市管理不断加强，数字化城管信息系统建成并投入运行;城市户外广告及店招门楣清理整治工作全面启动;拆除违法违章建筑18.9万平方米;中心城市公交完成国有化改革，新增公交车辆60台。和谐秀美乡村建设深入推进，36个乡镇、106个中心村镇村联动建设顺利推进，1447个村点建设任务全面完成，横峰县白沙岭分场等6个乡村成为全国首批“美丽乡村”创建试点村。

在全力支持“1+5”信江河谷城镇群建设政策推动下，探索打造内陆城镇化建设示范区工作正式启动。编制了“1+5”信江河谷城镇群建设规划，确立了“以中心城市建设为核心，以组团发展、产城互动、复合型、生态型为基本形态，努力构建一体化的城镇群综合体”的建设思路，启动了320国道南移、上饶至广丰、上饶至铅山快速通道建设，建立了城镇群产业发展和基础设施统筹推进的机制。

(七)深入推进县域经济发展，广丰、德兴跻身全国百强县做出新成绩

鼓励和推动县区经济各显神通、竞相发展，县域经济综合实力进一步增强。新增余干、玉山2个生产总值过百亿元县，新增余干、弋阳、婺源3个财政收入过10亿元县。通过积极争取，在原有3个县纳入原中央苏区振兴规划的基础上，新增横峰、弋阳两县纳入规划。万年县先行先试取得积极成效。玉山县工业化、城镇化“双轮驱动”，经济社会协调发展的经验得到省充分肯定。

在全力支持广丰、德兴跻身全国百强县政策推动下，两地加快了发展步伐。广丰县完成生产总值240亿元、财政总收入33亿元，入选“2013年度中国最具区域带动力中小城市百强县市”，成功创建“全国科技进步县”、“全国食品工业强县”等品牌。德兴市完成生产总值140亿元、财政总收入28亿元，获评“全国生态文明先进市”、“全国科技进步先进县市”等称号。

(八)扎实推进生态建设，再生资源循环经济产业园建设进入新阶段

坚持生态建设和环境保护同步推进。打造“森林城乡、绿色通道”精品工程82个，创建森林乡镇55个、森林村庄381个，完成造林36.3万亩。成功创建省级生态园林城市，并启动国家森林城市创建工作。“净空、净水、净土”环保专项行动扎实开展。黄金埠电厂脱硝、横峰永兴铜业烟气脱硫等18个列入国家、省重点减排计划的项目稳步推进，德兴、弋阳、铅山、横峰等国控、省控重金属污染防治重点区域的环境整治力度进一步加大。全面完成化学需氧量、氨氮、二氧化硫、氮氧化物减排任务，单位GDP能耗控制在省政府下达的计划之内。婺源县成为全省首批国家生态县，江湾镇入选全国首批美丽宜居小镇。德兴市、万年县纳入我国首个资源型城市可持续发展规划。

在全力支持再生资源循环经济产业园政策推动下，园区建设全面启动。产业园控制性详规已经编制完成，配套基础设施建设全面启动。项目招商工作正在抓紧推进。再生资源社区回收点建设以及乡镇回收站、回收中转站网络建设全面铺开。

(九)统筹推进社会事业发展，高校创建工作实现新突破

扎实推进民生工程建设。8大类96项民生任务全面完成。全市用于民生领域的财政支出增长22%，占公共财政预算支出的70.1%。全市城镇新增就业人员8.8万人。建立和完善了城乡居民大病保险等保障制度，在全省首创医疗保险“大病关爱”行动。保障性安居工程顺利推进，开工建设保障性住房5万套、建成3.2万套。科技创新取得重大进展，列入国家“863”计划项目实现了零的突破，袁隆平院士牵头的万年贡集团院士工作站正式设立，新增高新技术企业16家。教育优先发展战略得到落实，新(改、扩)建校舍80.6万平方米。公共卫生均等化服务得到加强，玉山县获得“国家卫生县城”称号。加强公共文化服务体系建设，文化事业进一步繁荣，文化惠民工程惠及城乡百姓，文化产业成为服务业重要增长点。成功举办了市第三届运动会，承办了中华龙舟赛鄱阳站等7项全国性体育赛事。低生育水平进一步巩固，出生人口性别比下降了1.1个基点。人民群众收入水平进一步提高，城镇居民人均可支配收入22195元，增长10%;农民人均纯收入7919元，增长13%。国防动员和民兵预备役工作进一步加强，夏季征兵工作圆满完成，以上饶命名的海军“上饶舰”正式入列。扎实推进平安上饶建设，安全生产、信访、社会治安、食品药品监管、应急管理等工作进一步加强，社会保持和谐稳定。

在全力支持高等院校创建政策推动下，江西医学院上饶分院被省政府正式批准设立为江西医学高等专科学校，并成为我省第一所与企业、医院、科研机构共建国际干细胞再生医学产学研基地的高校;上饶师院加快筹建教育、农业推广等五个学科硕士学位授予点;婺源茶校与江西农大成功开展合作办学;整合中师资源、筹建上饶幼儿师范高等专科学校进入筹划阶段。

此外，民族宗教、涉台事务、外事侨务、人民防空、气象水文、防震减灾、检验检疫、妇女儿童、新闻出版、广播电视、社科研究、志愿者服务、残疾人工作、无线电管理、红十字、地方志、档案、保密、工商、质监、石油、烟草、盐业、慈善、统计、审计、老龄等各项事业取得新成绩。

在努力做好经济社会发展工作的同时，政府自身建设也得到了加强。严格按照法定权限和程序行使权力、履行职责，自觉接受市人大及其常委会的法律监督、工作监督和政协的民主监督，主动向人大常委会报告工作，认真接受人大代表询问和政协委员意见建议，按期办复人大代表建议87件、政协提案188件，办结率100%，满意率97.4%。进一步加大简政放权力度，精简市级非行政许可审批项目30项，精简率达10%，项目审批时间平均缩短35%。扎实推进公共资源交易平台、统一电子监察平台、公共应急管理平台、中小企业信息化服务平台和电子印章系统建设。深入推进政府信息公开，主动公开信息18.4万条。严格落实“八项规定”，深入开展了“转作风、访民情、办实事，切实维护和保障群众利益”主题活动和“红包”专项治理活动，全市公务用车、公务接待费用和因公出国(境)人次均大幅下降。开展了民生资金管理使用情况监督检查等专项治理，严肃查处了一批损害群众利益的突出问题，坚决查办了一批违法违纪腐败案件。

各位代表：这些成绩的取得，是省委、省政府和市委统揽全局、正确领导的结果，是市人大、市政协有力监督、鼎力支持的结果，是全市人民齐心协力、共同奋斗的结果。在此，我代表市人民政府，向付出辛勤劳动的全市广大干部群众，向大力支持政府工作的人大代表、政协委员、各民主党派、工商联、无党派、各人民团体和社会各界人士，向关心支持上饶建设的离退休老干部、老同志，向为上饶改革发展稳定作出积极贡献的驻饶部队、武警官兵和公安民警，向中央和省驻饶单位及所有关心支持上饶建设发展的同志们、朋友们，向在我市创业的投资者、建设者，致以崇高的敬意和衷心的感谢!

回顾过去一年工作，我们清醒地看到，我市经济社会发展中还存在不少困难和问题，主要是：投资增幅有所回落，消费增长动力不足，经济运行存在较大的下行压力;实体经济运行困难，生产经营成本上升，部分企业效益下降或亏损;科技创新能力不强，产业结构不尽合理，结构调整任务艰巨;就业矛盾仍然突出，促进城乡居民持续增收难度较大;影响社会和谐稳定的因素仍然较多，社会治理还存在薄弱环节;政府工作与群众的期待还有差距，职能转变任务艰巨;一些政府工作人员服务意识和责任意识不强，作风建设、效能建设、廉政建设有待进一步加强。我们将采取有力措施，切实加以解决。

二、2014年工作安排

2014年是全面贯彻落实党的十八届三中全会精神、全面深化改革的第一年，也是完成“十二五”规划目标的关键一年。今年全市经济社会发展的总体要求是：全面贯彻落实党的十八大、十八届二中、三中全会、中央经济工作会议和省委十三届七次、八次全会精神，按照市委三届六次全会确定的“七大中心工作、一个重要保障”的总体部署，坚持稳中求进、稳中有为、稳中提质，坚持“四个同步推进”，打造“四个生态”，以全面深化改革为统领，以加快发展、提高经济发展的质量和效益为中心，着力放大高铁效应、打造区域中心、实现生态立市、建设幸福上饶，争当生态文明建设标兵、绿色崛起实践先锋，奋力迈出建设四省交界现代化区域中心城市和江西绿色崛起重要增长极的新步伐。

今年全市经济社会发展的主要预期目标是：生产总值增长10.5%，财政总收入增长13%，500万元以上固定资产投资增长18%，社会消费品零售总额增长13.5%，规模以上工业增加值增长12%以上，外贸出口增长7.5%，城镇居民人均可支配收入增长10%，农民人均纯收入增长11.5%，人口自然增长率、城镇登记失业率、居民消费价格总水平涨幅及节能减排各项任务严格控制在省政府下达的指标之内。

需要说明的是，这个预期目标是经过认真分析慎重提出的。一是充分考虑到今年全省发展的总体要求，体现了我市争先进位的愿望，在增幅的确定上略高于全省平均预期。二是充分考虑到今年国内外经济发展不稳定不确定因素依然较多，我国经济进入中高速增长阶段，预期目标不宜定得过高，否则不符合客观实际。三是充分考虑到与全省“十二五”规划目标的衔接，体现了省委、省政府对赣东区域发展目标的基本要求。

做好今年经济社会发展工作，总体上要把握好以下几点：一是坚持科学发展、加快发展，力争比全省发展速度更快一些。二是坚持把改革创新贯穿于经济社会发展各个领域各个环节，进一步增强发展的动力和活力。三是坚持产业优先，壮大实体经济，筑牢加快发展的基石。四是坚持项目带动，加快推进一批影响全局的重大项目建设。五是坚持生态立市，大力推进美丽上饶建设，不断巩固提升生态优势。六是坚持民生为重，积极推进基本公共服务均等化，让广大人民群众更多地共享改革发展成果。

今年重点做好八个方面的工作：

(一)抓好政府职能转变，全面深化经济体制改革

切实转变政府职能。完成市(县)政府机构改革。制定出台进一步加快县域经济发展的政策措施，深入推进扩权强县改革，给予县域经济最大自主发展空间，充分释放县域经济发展活力。深化行政审批制度改革，继续取消和下放一批行政审批事项，力争将上饶打造成为全国行政审批事项最少的地区之一。建立行政审批事项目录管理制度，并向社会公开。推进工商登记制度改革，实行“先照后证”和注册资本认缴登记制。制定政府向社会购买服务的具体措施，建立公平开放的公共服务购买制度。加快培育发展社会组织，推进行业协会、商会与行政机构脱钩。

深化财税体制改革。推进市县部门预算改革，加快建立规范统一的部门预算体系。稳步推进国库集中支付电子化管理改革。整合清理专项转移支付资金，提高均衡性转移支付比重。从紧编制“三公”经费预算，推进“三公”经费公开。继续推进“营改增”试点工作。把控制和化解政府性债务风险与深化投融资平台改革有机结合起来，进一步加强国有资产管理，做大做强国有投融资平台。

促进多种所有制经济共同发展。进一步完善国有资产管理体制，推进经营性国有资产集中统一监管。继续推进重点困难国有企业的改革。深化集体林权制度配套改革，全面完成国有林场改革试点工作。积极发展混合所有制经济，鼓励非国有资本参股政府性投资项目，探索组建混合型旅游产业投资集团。抓紧研究制定非公有制企业进入特许经营领域的具体办法，为非公有制经济健康发展营造良好的环境。实施“摇篮计划”，鼓励“饶商回归”，培育一批本土民营企业家。

深化土地利用管理制度改革。进一步规范征地程序，完善征地补偿办法，落实被征地农民的社会保障。按照国家部署，稳妥开展农村宅基地、农民房产确权登记发证和抵押、担保、转让试点工作，有序推进农村集体经营性建设用地改革。逐步建立城乡统一的建设用地市场。

(二)抓好合作、通关、物流等开放平台建设，加速融入东部沿海

加快合作平台建设。积极加入长三角城市经济协调会，主动接受长三角一体化和上海自贸区的辐射带动，加快建设赣浙闽皖开放合作区平台。支持鄱阳、余干、万年融入“南昌大都市区”,主动接受南昌核心增长极的辐射。鼓励企业实施“走出去”战略，支持晶科能源、百勤异VC钠等省级重点出口企业抢占国际市场。推行招商引资签约项目调度督查机制，确保重大招商项目早落地、早进资、早开工、早投产。全年力争新引进省外5000万元项目资金增长15%以上，实际利用外资增长10%以上。

构筑“大通关”格局。完善上饶与长三角经济区、海西经济区相关口岸的协作机制，大力推进通关、通检便利化。力争南昌海关驻上饶办事处升格为上饶海关。启动上饶综合保税区规划选址工作，加强申报沟通对接，争取早日获批并抓紧建设。

打造区域物流中心。推进上饶至宁波“五定班列”升级为双向对开“天天班”。抓紧编制上饶西货站物流园发展规划。争取开工建设海西(上饶)胜记国际物流城。积极培育壮大一批现代物流企业，构建开放性物流体系。

(三)抓好高铁经济试验区和空港经济区建设，加速构建综合交通枢纽

启动高铁经济试验区和空港经济区建设。完成高铁经济试验区规划，引导旅游、电子商务、文化创意等产业入区发展，先行推进高铁站房、旅游集散中心、万年青城等项目建设。鼓励婺源县、德兴市、玉山县、弋阳县、上饶县等5个设有高铁站的县(市)大力发展高铁经济。完成上饶三清山机场空港经济区规划，积极推进空港经济区道路、供电等基础设施建设，大力引进与航空产业有关的项目。

抓紧构建海陆空立体交通体系。加快杭南长高铁、合福高铁建设，确保杭南长高铁年底前竣工。开工建设九景衢铁路上饶段、坑口铁路物流专用线(上饶西货站)、高铁存车场(始发站)以及上饶高铁站房。启动皖赣铁路电气化改造、上饶—南平—宁德铁路及乐德支线延伸线前期工作。开工建设上饶至万年高速公路。继续抓好国省道和农村公路建设。加快推进三清山机场建设，确保主体工程基本完成。基本建成福州港上饶码头一期工程。

推进能源、信息、水利基础设施建设。推进黄金埠电厂二期、天然气利用工程等项目建设。推进万家屋顶光伏发电项目，开展鄱阳等绿色能源示范县建设。启动“智慧城市”建设，支持婺源开展试点工作。继续抓好4G网络、电子商务基础平台、数字档案馆、农村信息化工程建设。加快农田水利基本建设，全面开展小(2)型病险水库除险加固，继续推进中小河流治理，改造中低产田8万亩，建设高标准农田20万亩。启动城东堤改造、三江导排渠建设项目，积极争取大坳灌区、貊皮岭灌区列入国家建设计划。大力推进气象现代化建设，进一步提高防灾减灾能力。

(四)抓好园区经济和产业培育，着力推动工业升级

做强园区经济。统筹11个工业园区发展，防止低水平重复建设。完善园区规划，做好调园扩区工作。坚持集约化开发，提高园区土地利用率和产出率。在经开区规划建设总部经济产业园和飞地园区。完善重点项目领导挂钩负责制，全面推行招商项目代办制，积极帮助企业协调解决各类难题。继续支持经开区做大做强，支持广丰工业园区升格为国家级开发区。力争全市园区主营业务收入实现2400亿元以上，其中上饶经开区过700亿元，广丰工业园区过500亿元。

做大主导产业。抓住项目建设、培育领军企业、突破关键技术、创建特色基地等关键环节，加快改造传统产业，培育壮大新兴产业。继续大力发展光伏产业，加快推进晶科能源3000兆瓦光伏一体化等项目。继续大力发展光学产业，加快推进星汉光电一体化等项目。继续大力发展先进装备制造业，加快推进蓝途汽车产业园、中镁镁合金板材、中材机械系列输送设备、耐普矿机矿山设备及橡胶备件二期等项目。继续大力调整有色金属产业结构，重点抓好与德兴铜矿、永平铜矿等驻市省属企业的对接合作，加快推进江西乾海电磁线铜杆、铅山龙头岗铅锌矿开发等项目。继续大力发展黄金产业，加快推进黄金资源整合及开发步伐。力争全市规模以上工业增加值达到600亿元，主营业务收入达到2600亿元，全年新增规模以上工业企业100家。

(五)抓好龙头企业培育和农业综合体建设，加快发展现代农业

加快推进农业产业化经营。着力稳定粮食生产，严格耕地保护，提高粮食综合生产能力。加快网上稻谷现货交易平台建设，进一步扩大上饶粮食知名度。突出“特色、规模、品牌、效益、生态”，继续抓好特色农产品基地建设。大力培育一批农业龙头企业，扶持万年鑫星农牧股份有限公司、远泉实业集团等企业上市，扶持横峰县葛业开发有限公司等企业申报国家级龙头企业，力争全年销售收入过亿元的农业企业达50家。加快上饶出口茶叶质量安全示范区、万年供港生猪等农产品出口基地建设。继续抓好国家级和省级现代农业示范区创建。大力推进赣闽(台)农业合作示范区建设。规划建设以农业产业聚集为主，融合休闲观光、社会化服务、农产品展销为一体的农业综合体。加强农产品质量安全工作，健全食品安全追溯体系，从源头上确保广大人民群众“舌尖上的安全”。

抓紧构建新型农业经营体系。坚持和完善农村基本经营制度，加快构建以农户家庭经营为基础、合作与联合为纽带、社会化服务为支撑的立体式、复合型现代农业经营体系。建立农业风险保障体系，推动政策性“三农”保险。加大农业职业教育和技术培训力度，培养造就懂技术、会经营、善管理的新型农民队伍。

大力推进和谐秀美乡村建设。继续推进1421个新农村村点建设。推广婺源、玉山等地建设经验，加快推动50个试点乡镇镇村联动发展，加快改造“城中村”、“城郊村”。加强和创新农村社会管理，发展农村新型社区，健全农村留守儿童、留守妇女、留守老年人关爱服务体系，确保农村社会和谐稳定。

(六)抓好旅游经济和电子商务产业，不断提升现代服务业水平

全力提升旅游“第一品牌”。坚持发展“大旅游”，加快旅游业与相关产业融合发展，全面提升我市旅游业的产业化、市场化、国际化和现代化水平。力争全年接待游客达到6200万人次，旅游综合收入达到520亿元。加强旅游合作，推动编制赣东北旅游合作发展规划。深化旅游品牌创建，全力支持龟峰创建国家5A级景区，支持篁岭、严田古樟、云碧峰、灵山、神农源、大茅山、铜钹山等创建国家4A级景区，支持鄱阳湖国家湿地公园申请列入国家生态旅游示范区。开工建设上饶旅游集散中心和婺源旅游服务中心，抓紧建设和完善三清山枫林和银湖湾、紫湖、灵山、龟峰、德兴港首等一批重点旅游服务区或旅游换乘中心，加快推进余干瑞洪和大明湖旅游休闲度假区、鄱阳湖国际旅游度假区、婺源大型旅游文化实景演出、铅山河口古镇保护性开发、德兴温泉开发、三清书院、三清山中国道教文化园、大灵山文化园等重大项目。以灵山景区的建成开放为契机，完善城市旅游产品构建，逐步做热中心城市旅游。继续支持婺源、三清山加快发展，将婺源打造成全国旅游第一强县和国家乡村旅游度假实验区，将三清山打造成全国旅游十强景区和江西省旅游示范景区。加快智慧旅游建设，全面推行标准化行业管理。加大宣传力度，多领域、全媒体提升“上饶旅游”在国内外的知名度。

大力发展电子商务产业。制定出台电子商务产业扶持政策。加快建设电子商务产业园区，支持上饶信息产业园、玉山电子商务产业园分别创建国家级、省级电子商务示范基地。大力引进和培育电子商务龙头企业，推动“淘宝·特色中国”上饶馆、上饶慧谷、上饶电商谷等项目加快建设。扩大“电子商务进农村”工程试点范围，打造一批电子商务示范村。

繁荣发展商贸流通业。启动实施商务部第二批中小商贸流通企业服务体系建设试点城市项目，全力推进商贸流通业“2020”工程、菜市场提升工程、再生资源回收体系等重点项目建设，加快中心城市万力时代广场、万达广场、五洲国际商业城等大型商业综合体建设。完善政策措施，加快发展健康服务、养老服务、社区服务等生活性服务业。

加快发展现代金融业。积极争取银行、证券、保险等各类金融机构入驻我市，重点引进全国性股份制商业银行来我市设立分行，推动商业银行在我市人口较多、产业特色鲜明的县(市、区)设立支行。大力推进农信社改制组建农商行工作，实现村镇银行县域全覆盖。加大金融产品和服务创新力度，大力推动企业上市、推动中小企业集优票据、资产证券化等业务，提高直接融资占比。积极支持保险业拓展市场、深化改革、优化服务。继续推进社会信用体系建设，探索设立民间借贷登记服务中心，引导民间金融健康发展。加强小额贷款公司、融资性担保机构的监管，加大预防和打击非法集资活动的力度，切实防范金融风险。调整融资结构，努力化解实体经济融资难、融资贵问题，加大对重点项目、小微企业、涉农企业的资金支持力度。进一步拓宽融资渠道，扩大融资规模，力争全市金融机构本外币存款余额达到1955亿元，社会融资余额达到2000亿元。确保小微企业贷款增幅不低于各项贷款平均增幅，贷款总量不低于上年同期水平。

(七)抓好精细优美城市建设，努力打造内陆城镇化示范区

加大中心城市建设力度。启动《上饶市城市总体规划》、《上饶市综合交通规划》的修编工作，完成《上饶市城市风貌特色和慢行系统规划》等规划的编制。抓好高铁新城、空港新区、物流新城三个片区的开发，积极推进老火车站片区综合改造、上饶大道南北延伸、凤凰大道东西延伸、稼轩大道、天佑大道、天然气管网工程、城市引水工程、城区排污工程、三江口城市综合体等在建和续建项目。继续抓好公租房、安置房建设，加大棚户区改造力度，推进房地产市场健康发展。加快人防工程建设步伐，推动城市地下空间开发利用。

全面推进城市精细化、网格化、人性化管理。深化城市管理体制改革，推动管理重心下移，专业职能下沉，充分发挥街道、社区的作用。以国家森林城市、全国文明城市、全国卫生城市创建工作为契机，全面开展户外广告及店招门楣清理、违法违章建筑、环境卫生、交通秩序、地下管网普查及里弄小巷、饮用水源保护区环境等“六大整治行动”。

强力推动“1+5”城镇群建设。按照同城化、一体化、均等化的要求，抓紧各专项规划编制，制定引导基础设施建设、基本公共服务体系建设和社会发展领域建设方面的政策措施。建立健全“1+5”多层次合作协调会商机制，推动城镇群有序发展。积极稳妥推进行政区划调整，有序推动撤县设区工作。

协调推进县城和小城镇建设。按照以产立城、以城兴业、产城融合的思路，抓好鄱阳、余干、万年、德兴、婺源城镇化建设。全面放开农民进城落户限制，进一步降低农民进城的门槛。加大对农业转移人口进城就业的政策支持力度，把进城落户农民纳入城镇住房保障和社会保障体系。赋予中心镇部分县级经济社会管理权限，培育壮大33个省、市重点镇。

(八)抓好民生事业，积极推进基本公共服务均等化

进一步加强生态文明建设。实行最严格的源头保护制度、损害赔偿制度、责任追究制度，着力打造蓝天绿地、青山碧水美好家园。大力推进“森林城乡、绿色通道”工程建设，力争完成造林25.8万亩。加快推进城市污水处理厂西迁、城区空气PM2.5和水质自动监测站等重点环保项目建设。按照“布局优化、企业集群、产业成链、物质循环、集约发展”的要求，加快推进再生资源循环经济产业园建设。扎实推进重金属污染、工矿污染和农业面源污染综合防治。支持德兴、万年资源型城市转型发展，支持婺源争创国家生态文明先行示范区，支持鄱阳内珠湖列入全国优质湖泊生态环境保护项目。

进一步抓好各项民生工程。按照“保基本、兜底线、促公平、可持续”的要求，积极推进民生工程。深入实施高校毕业生、困难群体和农村剩余劳动力就业促进计划，力争新增城镇就业4.7万人，转移农村劳动力7.9万人，发放小额担保贷款7亿元。完善社会保障体系，力争城乡居民养老保险参保人数达到244万人，全年城镇居民基本医疗保险参保人数达到148.4万人，新农合参保人数达到560万人，确保工伤保险参保人数达到36万人，被征地农民养老保险参保人数达到7万人。逐步建立公开规范的住房公积金制度。提高社会救助水平，认真做好城市低保、农村低保、城镇“三无”特困群众的救助救济工作，切实抓好农村五保户的集中和分散供养工作。进一步实施优先发展城市公交战略，中心城市新增新能源公交车60辆。加大产业、金融、科教、旅游扶贫开发力度，增强贫困地区和贫困人口自我发展能力。切实抓好原中央苏区县、4个国定贫困县的扶贫开发和全市648个贫困村的整体推进工作。继续实施深山区、库区贫困群众移民扶贫搬迁工程，认真抓好21.4万水库移民的稳定和后期扶持工作。继续加快保障性住房建设，确保全年建设各类保障性住房5.1万套。

进一步加快科教文卫等社会事业发展。把创新驱动摆到更加突出的战略位置，努力建设创新型城市。深入实施科技创新“六个一”工程，建立和完善万年贡集团、江西(上饶)干细胞基础与临床应用研究所2个院士工作站以及德兴百勤异VC钠公司、晶科能源集团2个博士后科研工作站，加强高新技术企业、创新基金项目申报工作。加快教育事业发展，继续抓好高等院校创建工作，加大义务教育布局调整力度，严格执行义务教育阶段划片免试就近入学政策，组织开展教育对口支援工作，完善保障进城务工人员子女接受义务教育政策措施，加快推动青少年示范性综合实践活动基地和城东学校建设，新(改、扩)建中小学校舍40万平方米。重视和支持关心下一代工作，加强青少年社会主义核心价值体系教育。进一步加强卫生服务能力建设，筹建一所大型综合性医院，推进新农合支付方式改革，全面铺开新农合大病保险，巩固和完善儿童白血病、终末期肾病、重症精神病等20个重大疾病救助。加强文化建设，开展好第一次全国可移动文物普查，做好上饶美术馆、博物馆、群众艺术馆建设前期准备工作。继续抓好农村文化三项活动，丰富群众文化生活。积极发展文化创意产业。继续做好高水平体育赛事申办等工作，推动全民健身。继续把稳定低生育水平作为人口计生工作的首要任务，切实做好机构改革、计生政策调整等方面的衔接工作。落实双拥优抚政策，启动新一轮“双拥模范城(县)”创建工作。

进一步加强社会治理体系建设。建立健全重大决策社会稳定风险评估机制，建立畅通有序的诉求表达、利益协调、矛盾调处、权益保障渠道。改革信访工作制度，从源头上预防和化解社会矛盾。进一步落实安全生产工作责任，提升安全生产保障能力，杜绝重特大安全生产事故发生。认真做好消防安全工作。推进全市食品药品监管机构改革，建立覆盖生产、流通、消费全过程的监管制度体系。加强社会治理工作，大力推动“天网”、“地网”监控系统建设，严厉打击各种违法犯罪活动，确保一方平安。强化应急管理体系建设，提升突发事件预警、管控和处置能力。

各位代表!

新的一年，政府要履行好服务人民群众和经济社会发展的责任，必须进一步加强和改进自身建设。

进一步解放思想。坚持把解放思想贯穿于政府工作全过程，打破思维定势，冲破利益藩篱，突破狭隘视野，以更大决心和勇气破除思想观念和体制机制障碍，推动工作与时俱进、创新发展。严格依法行政。牢固树立法治理念，严格按照法定权限和法定程序行使权力、履行职责，更加自觉地运用法治思维和法治方式解决问题、处理矛盾、维护稳定。主动接受人大的法律监督、工作监督和政协的民主监督。自觉接受舆论监督和社会监督。加强监察、审计内部监督。普遍建立法律顾问制度。健全规范性文件审查制度，完善规范性文件、重大决策合法性审查机制，改革行政复议审理机制。进一步推进基层法制员制度建设，夯实依法行政基础。完善科学决策。广泛听取各民主党派、工商联、无党派人士和人民团体的意见和建议。认真办理人大代表建议和政协提案。建立健全决策咨询制度，加强智库建设。改进工作作风。认真贯彻落实中央“八项规定”，扎实开展群众路线教育实践活动，坚决纠正“四风”，努力提高公务员的素质。全面推进政府信息公开，让权力在阳光下运行。大力弘扬坚持不懈、一抓到底的务实精神，扑下身子抓落实，提高政府执行力和公信力。狠抓反腐倡廉。牢固树立廉洁政府理念，深入推进廉政风险防控管理，全面落实廉政建设责任制，加大预防和惩治腐败力度。全面推进电子监察、网上审批、公共资源网上交易，认真做好政府投资工程项目信息公开和“三公”经费公开工作，从源头上防治腐败。深入开展损害群众利益突出问题专项治理，坚决纠正部门和行业的不正之风，严肃查出各类违纪违法案件，切实做到干部清正、政府清廉、政治清明。

各位代表!全面做好今年的各项工作，任务艰巨，责任重大。让我们更加紧密地团结在以习近平同志为总书记的党中央周围，在省委、省政府和市委的坚强领导下，解放思想、锐意进取，攻坚克难、真抓实干，奋力迈出发展升级、小康提速、绿色崛起、实干兴赣的新步伐!
